# Genetic aspects of autism spectrum disorders: insights from animal models

**DOI:** 10.3389/fncel.2014.00058

**Published:** 2014-02-24

**Authors:** Swati Banerjee, Maeveen Riordan, Manzoor A. Bhat

**Affiliations:** Department of Physiology, Center for Biomedical Neuroscience, School of Medicine, University of Texas Health Science CenterSan Antonio, TX, USA

**Keywords:** autism spectrum disorder, synapse, animal models, genetics, epigenetics, environment, cell adhesion molecules, scaffolding proteins

## Abstract

Autism spectrum disorders (ASDs) are a complex neurodevelopmental disorder that display a triad of core behavioral deficits including restricted interests, often accompanied by repetitive behavior, deficits in language and communication, and an inability to engage in reciprocal social interactions. ASD is among the most heritable disorders but is not a simple disorder with a singular pathology and has a rather complex etiology. It is interesting to note that perturbations in synaptic growth, development, and stability underlie a variety of neuropsychiatric disorders, including ASD, schizophrenia, epilepsy, and intellectual disability. Biological characterization of an increasing repertoire of synaptic mutants in various model organisms indicates synaptic dysfunction as causal in the pathophysiology of ASD. Our understanding of the genes and genetic pathways that contribute toward the formation, stabilization, and maintenance of functional synapses coupled with an in-depth phenotypic analysis of the cellular and behavioral characteristics is therefore essential to unraveling the pathogenesis of these disorders. In this review, we discuss the genetic aspects of ASD emphasizing on the well conserved set of genes and genetic pathways implicated in this disorder, many of which contribute to synapse assembly and maintenance across species. We also review how fundamental research using animal models is providing key insights into the various facets of human ASD.

## INTRODUCTION

Autism spectrum disorders (ASDs) are a complex set of heterogeneous neurodevelopmental disorders categorized by a triad of key behavioral anomalies. Characteristic behavioral abnormalities consist of restricted interests accompanied by repetitive behavior, deficits in language and communication, and the inability to engage in reciprocal social interactions (Abrahams and Geschwind, 2008; Betancur et al., 2009; Levitt and Campbell, 2009; Peca et al., 2011b; Zoghbi and Bear, 2012). Autism is not a singular disease entity. The disorder encompasses a spectrum of wide ranging phenotypic manifestations which span from debilitating impairments to mild behavioral and personality traits. Therefore, autism is rightfully referred to as “autism spectrum disorders” (Persico and Bourgeron, 2006).

Autism spectrum disorder appears to be involved in early brain development. Obvious signs and symptoms show early onset within the first 3 years of life and persist into adulthood. According to the recent reports from the Center for Disease Control, an estimated 1 in 88 children has been identified with ASD. Interestingly, these disorders show a gender bias where males are affected almost five times more than females (http://www.cdc.gov/Features/CountingAutism/). ASD is among the most heritable disorders evidenced by family and twin studies with a concordance rate of 70–90% for monozygotic twins (Folstein and Rutter, 1977; Steffenburg et al., 1989; Bailey et al., 1995; Folstein and Rosen-Sheidley, 2001). Nevertheless, heritability in this case is more complex due to the differences in manifestations of its core symptoms, gradual changes over time, and differing degrees of response to interventions (Abrahams and Geschwind, 2008; Levitt and Campbell, 2009). 10–25% of ASD cases seem to have an underlying genetic disorder such as fragile X syndrome, tuberous sclerosis (TSC), and Rett syndrome (Betancur et al., 2009).

Recent studies have highlighted numerous potential risk factors that may contribute to ASD. These risk factors range from genetic, to epigenetic, to environmental factors. Detection of copy number variations (CNV), point mutations, and identification of rare variants in synaptic cell adhesion proteins and pathways are some of the ways researchers are providing insight into the pathophysiology of ASD (Sebat et al., 2007; Malhotra and Sebat, 2012; Zoghbi and Bear, 2012). It is worthwhile to note that the genes and the genetic pathways implicated in ASD, and the identification of any causal rare variants are accessible to modeling in experimental systems. Research findings both from studying human genetics and animal models of ASD suggest that disruption of synapse formation and stabilization processes is a key underlying feature in ASD etiology. Dysfunctions in the assembly or structure of transmembrane and scaffolding proteins needed for building and maintaining synapses, and disruption in cellular signaling pathways controlling synaptogenesis are major contributing factors in ASD.

A large portion of this review will emphasize the well-conserved sets of genes and genetic pathways implicated in ASD, many of which contribute to synapse assembly and maintenance across different species. Given the complexity and heterogeneity of this disorder, it has proved challenging to unravel the underlying causes of ASD from human clinical population alone. Nevertheless, numerous animal models have been utilized that have enormously contributed toward understanding specific aspects that constitute the spectrum of these disorders.

## HISTORICAL OVERVIEW OF AUTISM SPECTRUM DISORDERS

Leo Kanner, a psychiatrist, initially described autism well over half a century ago (Kanner, 1943, 1968, 1971). Studies on the relationship between autism and abnormal electroencephalogram were among the first to suggest autism as a disorder of brain function (Creak and Pampiglione, 1969). Despite these groundbreaking observations on autism, early identification of autism was marred by lack of adequate diagnostic criteria. It was not until the introduction of the concept of “autism triad” that highlighted the now well-established characteristics of impairment in social interaction, language and communication did Autism become a recognizable disorder. Since then the clinical conceptualizations of ASD have consistently evolved together with a steady rise in the number of ASD cases. Our current understanding of ASD is that of a complex neurological disorder that continues to challenge our ability to identify the underlying causal mechanisms.

## MANY FACETS OF AUTISM SPECTRUM DISORDERS

### GENETICS – COPY NUMBER VARIATION

Copy number variation is among the most widespread of structural variations in the human genome, and is increasingly being implicated as a major contributor to the pathophysiology of complex neurodevelopmental disorders (Sebat et al., 2007, 2009; Christian et al., 2008; Kumar et al., 2008; Marshall et al., 2008; Weiss et al., 2008; Bucan et al., 2009; Glessner et al., 2009; Merikangas et al., 2009; Luo et al., 2012; Malhotra and Sebat, 2012). CNVs largely comprise of duplications and deletions and can be *de novo* or familial. *De novo* CNVs are more prevalent in causing sporadic genomic disorders (McCarroll et al., 2008). The duplication or deletion events disrupt gene structure, expression, and function and are a common cause of developmental delay. Several studies suggest important role of CNVs in disease etiology, susceptibility, and inheritance (Beckmann et al., 2007; Estivill and Armengol, 2007). Large-scale genome-wide association studies are credited for detection of CNVs in rare cases of ASD (Ma et al., 2009a). Duplications and microdeletions in many loci are associated with ASD. Several studies identified duplication CNVs within 15q13 (Christian et al., 2008; Miller et al., 2009) and microdeletions at many loci in 16p11.2 (Sebat et al., 2007; Marshall et al., 2008; Weiss et al., 2008; Levy et al., 2011; Sanders et al., 2011), Williams syndrome locus 7q11.23, DiGeorge syndrome locus 22q11.2, 1q21.1, and Prader–Willi and Angelman syndromes at 15q11-13 (Glessner et al., 2009; Sanders et al., 2011).

Interestingly, genes associated with CNVs in ASD are involved in regulating synaptogenesis. Some of the genes include *NEUROLIGIN 4* (*NLGN4*; Jamain et al., 2003; Laumonnier et al., 2004), *SHANK3* (Durand et al., 2007; Moessner et al., 2007; Gauthier et al., 2009), *TBX1*, *PCDH10*, and *NHE9* (Morrow et al., 2008). Recent findings further reiterate a correlation between synapse formation and autism (Glessner et al., 2009; Mitne-Neto et al., 2011). In addition to these genes, some of the other genes recognized as risk factors in ASD include *NEUREXIN 1* (*NRXN1*; Kim et al., 2008; Bucan et al., 2009), *SHANK2* (Berkel et al., 2010), CNTN4 (Fernandez et al., 2004), *CNTNAP2* (Bakkaloglu et al., 2008; Penagarikano et al., 2011), *DPYD* and *DPP6* (Marshall et al., 2008); NLG1 (Glessner et al., 2009) and SYNGAP1, DLGAP2 (Pinto et al., 2010). A detailed list of genes, their potential functions and genetic pathways linked to ASD are summarized in **Table 1**.

**Table 1:** Conserved genes implicated in ASD.

Gene	Protein description	Nature of abnormality	Reference
NRXN1	Transmembrane	Mutation, CNVs	Feng et al. (2006)
NRXN2	Transmembrane	Mutation	Arstikaitis et al. (2011)
NRXN3	Transmembrane	Mutation	Vaags et al. (2012)
NLGN1	Transmembrane	Genetic association	Glessner et al. (2009)
NLGN3	Transmembrane	Mutation	Jamain et al. (2003)
NLGN4	Transmembrane	Mutation, CNVs	Jamain et al. (2003)
CNTN3	Ig-CAM	Mutation, CNVs	Morrow et al. (2008)
CNTN 4	Ig-CAM	Mutation	Roohi et al. (2009)
CNTNAP2	Transmembrane	Mutation, genetic association	Arking et al. (2008)
NrCAM	Ig-CAM	Genetic association	Marui et al. (2009)
CDH9/10	Transmembrane	Genetic association	Bucan et al. (2009)
CDH18	Transmembrane	Chromosomal abnormality	Marshall et al. (2008)
PCDH9	Transmembrane	Mutation	Marshall et al. (2008)
PCDH10	Transmembrane	Mutation	Morrow et al. (2008)
PCDH19	Transmembrane	Mutation	Dibbens et al. (2008)
SHANK1	Scaffolding	Mutation	Sato et al. (2012)
SHANK2	Scaffolding	Mutation	Berkel et al. (2010)
SHANK3	Scaffolding	Mutation	Durand et al. (2007)
DLG4 (disk large homolog 4)	Scaffolding	SNPs	Feyder et al. (2010)
HOMER1	Scaffolding	Mutation	Kelleher et al. (2012)
cAMP-GEF (guanine exchange factor)	Cytoskeletal	Mutation	Bacchelli et al. (2003)
RELN (Reelin)	Secreted	Genetic association	Persico et al. (2001)
EN2 (Engrailed 2)	Transcription factor	Genetic association	Gharani et al. (2004)

### EPIGENETICS

Epigenetic mechanisms underlie several human neurodevelopmental disorders. Genomic imprinting, epimutations, DNA methylation, and histone modifications are all examples of epigenetic mechanisms linked to the development of certain disorders. These mechanisms involve modifications of nucleotides or chromosomes without altering the genetic sequence (Zoghbi, 2003; Egger et al., 2004). Thus causing modifications in gene expression that may increase the likelihood of developing a particular disease. Epigenetic mechanisms are believed to function at the interface between genetic and environmental factors (Jiang et al., 2004; Qiu, 2006). Studies linking these two factors are gaining importance for understanding the etiologies of complex disorders and could play a role in the development of ASD.

While epigenetic mechanisms are implicated in the development of many disorders, they are also an intrinsic phenomenon for normal brain development. Genomic imprinting is an example of an epigenetic mechanism that occurs normally throughout life. This is when one of the two parental alleles for an imprinted gene becomes inactive due to DNA methylation resulting in monoallelic gene expression. This phenomenon occurs quite frequently in humans but was also discovered in fungi, plants, and other animals (Martienssen and Colot, 2001; Jiang and Kohler, 2012). Using genome-wide scans, several areas on chromosomes known as, hot spots for genomic imprinting, were located on loci 7q and 15q (Reik and Walter, 2001; Luedi et al., 2007). Interestingly, these loci are highly affected in individuals with ASD (International Molecular Genetic Study of Autism Consortium, 2001; Lamb et al., 2005). Several studies have linked duplication or deletion events on the active chromosome to ASD (Cook et al., 1997; Schroer et al., 1998; Koochek et al., 2006). Individuals with Angelman syndrome (Mabb et al., 2011; Huang et al., 2012) and Prader–Willi syndrome (Miyake et al., 2012) show a defect in the active allele that leads to loss of gene expression. Such correlations provide compelling evidence for the role of genetic and epigenetic mechanisms in the etiology of ASD.

Additionally, DNA methylation is an important basic step in epigenetic gene control. Methyl-CpG binding proteins bind to the methylated DNA regions to control gene expression. Mutations in methyl-CpG binding protein 2 (MeCP2) cause Rett syndrome which shows characteristic autistic-like behavior in addition to seizures, ataxia, and stereotypic hand movements (Amir et al., 1999). More recently, MeCP2 was shown to regulate several genes involved in synaptic plasticity, neuronal cell proliferation and neuronal transcription factors including: brain-derived neurotrophic factor (BDNF), distal-less homeobox 5 (DlX5), and insulin-like growth factor binding protein 3 (IGF3; Chen et al., 2003; Martinowich et al., 2003; reviewed in Miyake et al., 2012). Thus, epigenetic misregulation of synaptic genes could potentially contribute to ASD (Beaudet, 2007). Yet another set of studies suggest that extrinsic factors like the environment can alter epigenetic make up leading to defective neuronal functions (Jessberger et al., 2007; Ma et al., 2009b).

### ENVIRONMENT AND OTHER FACTORS

Environmental contributions and other modulating factors are emerging as potential risk factors for ASD. Heavy metals, parental age, immunological proteins, environmental pesticides and insecticides, and food contaminants are thought to act as modulators of ASD (Durkin et al., 2008). These factors could contribute toward an increase in the prevalence of ASD but may not be sufficient to cause ASD. Nonetheless, a major challenge is to identify environmental factors relevant to ASD that could influence susceptibility, severity, and intervention outcomes. Heritable genetic vulnerabilities can magnify the adverse effects triggered by environmental factors. If both genes and environment converge, a resulting dysfunction of neurotransmitters and signaling pathways could take place at key developmental time points (Pessah et al., 2008). The toxicological literature point toward several environmental chemicals of concern to human health that can either directly or indirectly affect signaling pathways impaired in ASD.

Prenatal exposure to certain pesticides and insecticides are known to inhibit acetylcholine (ACh) and γ-aminobutyric acid (GABA; Shelton et al., 2012). Studies show these neurotransmitter systems are altered in a subset of autistic individuals. Similarly, environmentally induced alterations in calcium signaling pathways caused by organic pollutants, impact a broad range of neurotransmitter systems like the cholinergic, GABAergic, and dopaminergic systems (Pessah et al., 2008; Corrales and Herbert, 2011). In addition to disruptions in important neuronal signaling pathways, pesticides can cause oxidative stress, neuroinflammation, and mitochondrial dysfunction, all contributors to neuronal cell-death and dysfunction (Herbert, 2010; Shelton et al., 2012). Furthermore, cytokine-mediated influences and immune-related proteins are also listed as modulating factors for ASD (Ashwood et al., 2011; Onore et al., 2012). Families with ASD often show clustering of autoimmune disorders (Croen et al., 2005; Currenti, 2010). Several reports indicate the presence of serum antibody reactivity against human cortical and cerebellar regions of the brain in autistic patients (Silva et al., 2004). This process is thought to begin *in utero* and is associated with placental transfer of maternal autoantibodies to neuronal proteins potentially leading to neuronal dysfunction.

## ANIMAL MODELS OF AUTISM SPECTRUM DISORDERS

Autism spectrum disorder is a complex disorder with no singular pathology and because of this a collective and collaborative approach is the key to understanding its etiology and design of rational interventions. Studies in animals are aimed at modeling the core phenotypes associated with ASD, including communication and social impairments, restricted interests, and repetitive behaviors in an attempt to uncover the mechanisms that underscore the entire spectrum of the disorder. In this section, we will uncover the wide range of both invertebrate and vertebrate model systems utilized by researchers that have collectively made significant contributions toward understanding the mechanisms that underlie ASD (summarized in **Table 3**).

### NON-HUMAN PRIMATES

One of the animal models largely believed to help bridge the gap between humans and lower vertebrate systems is the non-human primate (NHP) model. NHP model is relevant for understanding ASD due to its high degree of correspondence to human behavior and their striking homology in the anatomy of neural circuits that mediate social behavior (for review, see Ongur and Price, 2000; Watson and Platt, 2012). Some of the behavioral correlates that NHPs have with human behavioral deficits seen in ASD include repetitive behaviors (Lutz et al., 2003; Alarcon et al., 2008), social communication (Ghazanfar and Santos, 2004), and their ability to follow other’s gazes, a tendency that is compromised in Autism. For example, ablation studies in NHPs especially of the superior temporal sulcus region reveal difficulties in responding to social cues like eye gaze (Campbell et al., 1990). The lesion model involving the amygdala in NHPs is used to study alterations in socio-emotional behaviors (Amaral et al., 2003). Some papers speculate on the involvement of mirror neurons in the development of ASD (Oberman et al., 2005). In early childhood development, mirror neurons may play a key role in mimicking behaviors, actions, and language. A failure in the development or proper organization of mirror neurons might be linked to some of the behavioral phenotypes associated with ASD (Williams et al., 2001). NHP, like humans, possess mirror neurons and their use as a model system could provide some insight into the involvement of mirror neurons in ASD. NHP models are also used to investigate immunological factors in the etiology of ASD. Autoantibodies present in children with ASD have prompted investigators to analyze the affects of maternal IgG antibodies on the fetal brain during gestation. Injections of IgG antibodies from human mothers who had multiple children with ASD to pregnant rhesus monkeys resulted in abnormal stereotyped behaviors in offspring, and increased activity of offspring compared to controls (Martin et al., 2008). Although use of NHP model has the capability of contributing to some of the more behavioral aspects of ASD research, the absence of genetic knockouts in NHPs modeling ASD together with the careful considerations of ethical implications of NHP research tend to pose limitations that can be better addressed using rodents and invertebrate model systems.

### RODENTS

Mouse models recapitulating symptoms of ASD through selective manipulations of genes and neural circuitry is a much more amenable model system compared to NHP models. Currently, there is a sizeable number of autism mouse models available made possible due to generation of specific gene knockouts; mutations in these genes are thought to contribute to ASD together with the emergence of CNVs and high-end genome-wide sequencing studies. Mouse models of human disorders have limitations in recapitulating the entire phenotypic spectrum (Arguello and Gogos, 2006). The validity of mouse models of human disorders are based on three criteria: (i) construct validity as provided by knock outs that carry a mutation in a gene that is affected in the human disorder (Peca et al., 2011a), (ii) face validity as reflected in animals that bear many of the core and ancillary physical or behavioral resemblances to the human disorder (Crawley, 2004), and (iii) predictive validity, which by far, is the most challenging to accomplish and indicates a similar response in the mouse model to an intervention that is known to be effective in human patients with that disorder.

Some of the mouse models representing syndromic forms of ASD include mice modeling Phelan–McDermid syndrome (SHANK3; Bangash et al., 2011; Peca et al., 2011a), Rett syndrome (MeCP2; Shahbazian et al., 2002; Moretti et al., 2006), fragile X syndrome (FMR1; Ronesi et al., 2012), Timothy syndrome (TS; CACNA1C; Bader et al., 2011), and others (see also **Table 2**). Neuroligin 3 knock out mice serve as a model for non-syndromic autism (Baudouin et al., 2012). Other examples of mouse models to study characteristics of ASD include Purkinje-specific knock out of TSC1 (Tsai et al., 2012), chromosome-engineered mouse model for human 15q11-13 (Nakatani et al., 2009), model for 16p11.2 lesion found in autism (Horev et al., 2011), 22q11.2 mice lacking PTEN (Zhou et al., 2009), CNTNAP2 (Penagarikano et al., 2011), SHANK2 (Won et al., 2012), and SCN1A (Han et al., 2012). The impressive array of mouse models displaying behaviors that are reflective of the human behavioral and cognitive ASD symptoms is highly informative. On the other hand, the behavioral phenotypes between mouse models with ablation of the same gene show variations based on either their genetic backgrounds (Crabbe et al., 1999), or how individual laboratories conduct their behavioral assays further underscoring the impact of the genetic background or the local environment on the displayed phenotypes. In any case, a complete understanding of the similarities and differences in the behavioral phenotypes across the ASD mouse models will provide key insights into the underlying neural circuitry behind these behaviors.

**Table 2:** Genetic syndromes with ASD-related phenotypes.

Syndrome	Chromosome	Genes	Reference
Angelman	15q11	Ube3A	Nurmi et al. (2001)
Phelan–McDermid	22q13	Shank3	Durand et al. (2007)
Rett	Xq28	MeCP2	Amir et al. (1999)
Tuberous sclerosis	9q34	TSC1	Baker et al. (1998), Smalley (1998)
	16p13	TSC2	
Timothy	16p13	CACNA1C	Splawski et al. (2004)
Fragile X	Xq27	FMR1	Rogers et al. (2001)
Cortical dysplasia-focal epilepsy syndrome	7q35	CNTNAP2	Arking et al. (2008)
Smith–Lemli–Opitz	11q13	DHBR7	Tierney et al. (2001)

Other emerging rodent models of ASD include rat and prairie vole (McGraw and Young, 2010). Rats that are injected with valproic acid (VPA) serve as an environmentally triggered model of autism and this method has emerged as a new way to study ASD in rats (Rodier et al., 1997). VPA injected to gestational mothers before neural tube closure causes autistic-like phenotypes in offspring such as a reduction in the number of cerebellar Purkinje cells and disruption in inhibitory circuits (Gogolla et al., 2009). Furthermore, these animals show similar behavioral phenotypes associated with autism including lower sensitivity to pain and higher sensitivity to non-painful stimuli, repetitive behaviors, hyperactivity, and decreased number of social behaviors. They also show delayed mental impairments and lower body weight (Schneider and Przewlocki, 2005; Favre et al., 2013). Prairie voles also generated interest in the area of ASD because of their ability to form lasting social bonds and their nurturing behavior. Impaired social behaviors and deficits in various aspects of social cognition are some of the signature of ASD in humans. Thus, development of genetic, molecular, and genomic tools in prairie vole will likely be useful in basic and translational research that may be relevant to ASD (McGraw and Young, 2010).

### ZEBRAFISH

Zebrafish are widely used as a model for studying vertebrate development and although not as popular as the mouse model for studying ASD, zebrafish are being used to dissect the genetic basis of autism due to a multitude of genetic techniques available. These include lineage tracing using fluorescent tracers or labeling cells with lipophilic dyes, loss of function analyses using chemicals, transposable elements, and gain of function assays such as those involving microinjection of synthetic mRNA. In addition, morpholino technology is widely used as an efficient reverse genetic approach to understand gene function (Tropepe and Sive, 2003). Furthermore, fast oogenesis and embryogenesis, and high fecundity allows for rapid experimental assays in this model. Transparency and external development of embryos allows for the study of growth and development of cells and tissues in live embryos (Tropepe and Sive, 2003). Additionally, zebrafish are an excellent model for use in carrying out genetic screens to identify new genes of interest and are useful in designing genetic screens to uncover specific enhancers or suppressors of particular phenotype (Dooley and Zon, 2000). These screens identified several candidate genes, such as Reelin and MET that confers susceptibility to human ASD in zebrafish (Rice and Curran, 2001). The presence of structurally and functionally homologous regions in zebrafish brain, which are perturbed in human autistic patients, is another avenue that zebrafish researchers are taking advantage of to study brain development and neuronal connections. Although zebrafish is an excellent model to study some of the genetic aspects of ASD, the behavioral phenotypes associated with ASD are difficult to recapitulate (Tropepe and Sive, 2003). Thus, other model systems might be useful in studying some of the behavioral phenotypes associated with ASD.

### SONGBIRDS

Songbirds can be used both as a molecular and a behavioral model for understanding the etiology of ASD. Songbirds are socially sophisticated and display characteristically human traits like monogamy and cultural inheritance while demonstrating the ability to learn vocalizations (Clayton et al., 2009). Vocal learning is an important element of language. Impairments in vocal and language learning are some of the core deficits in autism. Thus, understanding vocal learning through songbirds has emerged as an important model to study some aspects of ASD.

Speech in humans and bird songs display striking parallels in that both seem to have a critical developmental time window for learning, a homologous underlying neural circuitry involving a loop between the cerebral cortex, basal ganglia, and thalamus, and a role for social influences in the learning of vocalizations (Panaitof, 2012). Studies from songbird indicate that CNTNAP2, which is implicated in human ASD and is enriched in human language related neural circuits (Alarcon et al., 2008), might play a role in vocal communication in songbirds as well (Panaitof et al., 2010). Similar to developing human brain where CNTNAP2 shows a gradient distribution in frontal cortical areas, Cntnap2 expression is either enhanced or reduced in key song control nuclei in songbird brain. In the absence of an animal model that can address language deficits, songbird model may prove useful for further exploration of the cellular and molecular mechanisms underlying the homologous neural circuitry that underscore language development in humans. Recently, using microarray and *in situ* hybridization analyses, large databases have been compiled that reveal expression patterns of certain genes in specific regions of the brain (Warren et al., 2010). Expansion on the molecular aspects of these observations has further increased the validity of behavioral songbird research. While linking behavior and genetics in songbirds is a tall order, it might still provide important clues about neuronal circuitry and language acquisition in the complex and heterogeneous nature of ASD and its behavioral manifestations.

### INVERTEBRATE MODELS

Despite being millions of years apart on the evolutionary scale there is a surprising degree of genetic conservation between invertebrates and humans. Invertebrate models have made seminal contributions toward a basic understanding of human neurological disorders that are hard to ignore. One such classic invertebrate model for studying human neurodevelopmental disorders is the fruit fly, *Drosophila*. The fruit fly has proven to be an important model time and again to study various disorders where a single, causative genetic defect has been identified in Rett syndrome (Cukier et al., 2008), fragile X syndrome (Morales et al., 2002), and Angelman syndrome (Wu et al., 2008; see also **Table 2**). *Drosophila* studies have advanced our fundamental understanding of some of these human disease gene functions, which show features of ASD. Recent studies in *Drosophila* have started to unravel some of the key genes, such as Neurexin 1 (Li et al., 2007; Zeng et al., 2007), Neuroligin 1 (Banovic et al., 2010), and Neuroligin 2 (Chen et al., 2012), which are the fly homologs of human NRXNs and NLGNs, respectively, that are implicated in ASD (De Jaco et al., 2005; Sudhof, 2008). With the unmatched power of *Drosophila* genetics and the potential of carrying out large scale screens using the sophisticated genetic tools available, *Drosophila* will undoubtedly continue to provide key mechanistic insights to dissect the genetic basis of ASD and likely facilitate the design of therapeutics. Apart from *Drosophila*, other invertebrate models that are being used to study aspects of ASD are *C. elegans* (Calahorro and Ruiz-Rubio, 2011, 2012) and *Aplysia* (Choi et al., 2011; Ye and Carew, 2011).

A recent study in *Aplysia* showed that trans-synaptic Neurexin–Neuroligin interactions govern synaptic remodeling and regulates signaling required for the storage of long-term memory, including emotional memory, an ability that is affected in ASD patients (Choi et al., 2011). This study showed that long-term facilitation and associated pre-synaptic growth were compromised when neurexin or neuroligin was depleted from pre- and post-synaptic machineries. In addition, an introduction of R451C mutation of NLGN3 associated with human ASD blocked both intermediate- and long-term facilitation in *Aplysia* (Choi et al., 2011).

Another genetically tractable animal model is the nematode, *C. elegans*, which are utilized to understand the underlying mechanisms and abnormalities in neuronal synaptic communications in complex human neurological disorders like Alzheimer’s and ASD (Calahorro and Ruiz-Rubio, 2012). *C. elegans* have orthologs for ASD-related genes such as *NLGNs*, *NRXNs*, and *SHANK*. Recent reports highlight behavioral phenotypes in *C. elegans* reminiscent of ASD following removal of neuroligin homolog, nlg-1 (Hunter et al., 2010; Calahorro and Ruiz-Rubio, 2012) and subsequent functional phenotypic rescue by human NLGN1 (Calahorro and Ruiz-Rubio, 2012). Additionally, trans-synaptic NRX-1 and NLG-1 in *C. elegans* are found to mediate retrograde synaptic inhibition of neurotransmitter release at the neuromuscular junction (Hu et al., 2012) further underscoring the function of these molecules in synaptic modulation.

Thus determining the biological underpinnings of ASD will require a concerted effort involving studies from human clinical populations and several different animal models. This effort will provide complementary and critical insights toward understanding the complex and unknown ASD etiology. Since testing the causality and exploring the molecular and cellular mechanisms of ASD are either severely limited or off limits in human populations, research using various animal models will provide clues to the range of functional deficits that cause the disorder and may even hint at the underlying neural circuits that drive the behavioral deficits.

## GENES IMPLICATED IN ASD

**FIGURE 1:**
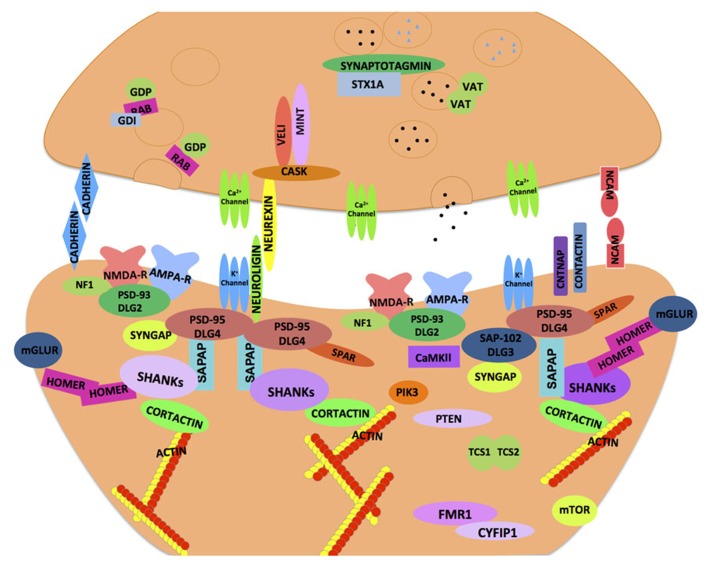
**Synaptic proteins implicated in neurodevelopmental and neuropsychiatric disorders.** A schematic illustration of an ensemble of pre- and post-synaptic proteins. The majority of these proteins are highly conserved across species, and thought to confer susceptibility to a host of neurodevelopmental and neuropsychiatric disorders including ASD. The cellular machinery of synapses is comprised of transmembrane heterophilic (such as Neurexin and Neuroligin) and homophilic cell-adhesion molecules (such as Cadherins and NCAM), cytoplasmic scaffolding proteins (such as PSD-95, Cask, and Shank) and cytoskeletal proteins (such as Homer and Cortactin) that link transmembrane and membrane-associated protein complexes with the underlying actin cytoskeleton. One of the emerging models in ASD is based on synaptic dysfunction in a molecular pathway that is orchestrated by trans-synaptic Neurexin–Neuroligin-dependent proteins complexes. This molecular assembly aligns the pre- and post-synaptic apparatus facilitating functional activation and modulation of ion channels that are in proximity to the neurotransmitter containing synaptic vesicles on the pre-synaptic side. These protein complexes recruit other proteins both pre- and post-synaptically and help organize functional neural networks. The cytoskeletal scaffolding protein Cask is one such notable protein which binds to the C-terminus of Neurexin. At the post-synaptic density, Neuroligin binds to PSD-95, other molecules such as PSD-93, SAP97, and the SAPAP family of proteins as well as the Shank family of scaffolding proteins help orchestrate the post-synaptic area. Homer and Shank function is thought to stabilize the post-synaptic density and serve as a platform to incorporate the post-synaptic receptors (such as NMDAR, AMPAR, and mGluR) into the machinery. The synaptic dysfunction in ASD may occur at multiple levels whereby failure to organize proper protein–protein interactions at the synapse may compromise neuronal functions (*refer text for more details*).

With the growing repertoire of synaptic genes implicated in ASD, it is becoming increasingly clear that synaptic dysfunction at multiple levels may underlie ASD. Synapses comprise of pre- and post-synaptic elements (**Figure 1**; see also review by Delorme et al., 2013) like synaptic cell adhesion molecules (CAMs), ion channels, neurotransmitter receptors, scaffolding and cytoskeletal proteins that work harmoniously to provide synaptic structural integrity and functionality (refer **Tables 1** and **4**). Perturbations in synaptic assembly or function are commonly reported in many neuropsychiatric disorders (Blanpied and Ehlers, 2004).

**Table 3:** Phenotypic analyses and relevant animal models of ASD.

Phenotype	Animal models								
	Non-human primate	Mouse	Rat	Prairie vole	Songbird	Zebrafish	*Drosophila*	*Aplysia*	*C. elegans*
Genetic analyses		+	+			+	+	+	+
Molecular analyses		+	+		+	+	+	+	+
Hyperactivity and repetitive behavior	+	+	+						
Social communication	+	+	+	+	+				
Cognition		+	+						
Impaired vocalization		+			+				

**Table 4:** Receptors, transporters, and channel proteins in ASD.

Gene	Gene name	Genetic abnormality	Reference
AGTR2	Angiotensin II receptor, type 2	Mutation	Vervoort et al. (2002)
ADRB2	Adrenergic β-2 receptor	Genetic association	Cheslack-Postava et al. (2007)
AVPR1A	Arginine vasopressin receptor 1A	Mutation, genetic association	Yirmiya et al. (2006)
DRD3	Dopamine receptor D3	Genetic association	de Krom et al. (2009)
ESRRB	Estrogen-related receptor β	Genetic association	Wang et al. (2009)
GABRB3	GABA A receptor, β3	Genetic association	Cook et al. (1998)
GABR4/GABRB1	GABA A receptor, α4/β1	Genetic association	Ma et al. (2005)
GRIP1	Glutamate receptor interacting protein	Mutation, genetic association	Mejias et al. (2011)
GRIK2	Glutamate receptor, kainate 2	Genetic association	Jamain et al. (2002)
GRIN2A	Glutamate receptor, ionotropic, *N*-methyl D-aspartate 2A	Mutation	Barnby et al. (2005)
GRIN2B	Glutamate recepto14r, ionotropic, *N*-methyl D-aspartate 2B	Mutation	O'Roak et al. (2011)
GRID1	Glutamate receptor, δ1	Mutation	Glessner et al. (2009)
GRID2	Glutamate receptor, δ2	Mutation	Schaaf et al. (2011)
GRM5	Glutamate receptor metabotropic 5	Mutation	Iossifov et al. (2012)
OXTR	Oxytocin receptor	Genetic association	Wu et al. (2005)
SL6A4	Solute carrier family 6 (serotonin transporter)	Genetic association	Sutcliffe et al. (2005)
SLC25A13	Solute carrier family 25 (aspartate-glutamate carrier)	Genetic association	Ramoz et al. (2004)
CACNA1C	Calcium channel, α1B subunit	Mutation	Splawski et al. (2004)
SCN1A/SCN2A	Sodium channel, α subunit	Mutation	Weiss et al. (2003)
KCNJ10	Potassium channel subfamily	Genetic association	Sicca et al. (2011)

### CELL ADHESION MOLECULES

#### Neuroligins

Neuroligins (NLGNs) are post-synaptic CAMs localized at glutamatergic or GABAergic synapses (Song et al., 1999; Varoqueaux et al., 2004). NLGNs have a large extracellular cholinesterase-like domain, and a small intracellular domain with PDZ-binding motif. There are five NLGNs, which include two X-linked genes (*NlgN3* and *NlgN4X*) and one Y-linked (*NlgN4Y*). The identification of ASD-linked mutations in NLGN3 and NLGN4X was an important finding that implicated these genes in the etiology of ASD (Jamain et al., 2003) and linked ASD to molecules with synaptic function. *In vitro* studies using mutant forms of *NlgN3* and *NlgN4X* showed retention of the mutant proteins in endoplasmic reticulum resulting in reduced cell surface binding to Neurexin (NRXN; Chih et al., 2004; Comoletti et al., 2004; Boucard et al., 2005). Some of the mutations of NLGNs seen in ASD patients were generated in mice and other model systems. For example, *NlgN3* R451C knock-in mice showed challenged social interactions, enhanced inhibitory synaptic transmission, and altered spatial learning abilities (Jamain et al., 2003; Tabuchi et al., 2007; Chadman et al., 2008). This arginine to cysteine point mutation at the analogous position as the human NLGN3 R451C was recently made in *Aplysia* revealing abnormal synaptic facilitation (Choi et al., 2011). *NLGN3* knock out mice showed reduced ultrasound vocalization together and a lack of social novelty preference (Radyushkin et al., 2009). *NlgN4* knock out mice displayed reduced reciprocal social interactions and vocalizations consistent with observations in human ASD patients (Jamain et al., 2008). Studies on *NlgN1* knock out mice showed impaired NMDA receptor signaling, while *NlgN2* knock out mutants revealed reduced inhibitory synaptic transmission (Chubykin et al., 2007). Studies in vertebrate and invertebrates alike have now established that NLGNs are essential for proper synapse maturation, maintenance, and function as opposed to initial synapse formation (Chih et al., 2005; Varoqueaux et al., 2006; Sudhof, 2008; Banovic et al., 2010; Chen et al., 2012).

Recent studies on null mutants of *Drosophila neuroligin 2* (*dnlg2*), which is the fly homolog of human *NlgN3* showed reduced synaptic bouton numbers and synaptic transmission (Chen et al., 2012). Interestingly, *dnlg2* is required both pre- and post-synaptically for proper synapse structure and function. Another study in *C. elegans* reported presence of Neuroligin at both pre- and post-synaptic regions (Feinberg et al., 2008). These studies highlight the exceptions to the traditional role and localization of NLGNs at the post-synaptic terminals as seen at most vertebrate synapses. It also raises interesting questions about how synaptic organization might be fine tuned, and how signaling pathways might regulate the expression of pre- and post-synaptic proteins during synaptic development and function. Thus, studies on NLGNs using various model systems will provide key insights into how these synaptic CAMs are involved in human ASD.

#### Neurexins

Neurexins (NRXNs) are predominantly presynaptic CAMs (Ichtchenko et al., 1995), although they were also reported to be expressed post-synaptically (Taniguchi et al., 2007). There are three *Nrxn* genes, *Nrxn1*, *Nrxn2*, and *Nrxn3*, each of which encode α- and β-isoforms. The α-Neurexin extracellular domain consists of six LNS domains interspersed by three EGF-like repeats and interacts with various proteins in the synaptic cleft. Mouse knock out mutants of individual Nrxns do not show gross abnormalities in synaptic ultrastructure or in synapse number while triple α-Nrxn knock out mice die prenatally due to respiratory complications. These mice show impaired synaptic transmission, but not synapse formation suggesting that like their Nlgn ligands, α-NRXNs are required for proper synaptic maintenance and function, and not initial synapse formation (Missler et al., 2003). Pre-synaptic calcium channel function is also disrupted in *α-Nrxn* knock out mice. Interestingly, compared to three NRXNs in mammals, *Drosophila *has a single *neurexin-1* (*dnrx*) gene which like its vertebrate counterparts is pre-synaptic and is required for proper synaptic growth and neurotransmission (Li et al., 2007).

NRXN1 has emerged as a strong candidate in ASD since the identification of overlapping *de novo *deletions in *Nrxn1* in individuals with ASD. Although rare, missense mutations (Feng et al., 2006; Kim et al., 2008) and deletions, and chromosomal aberrations in the *NRXN1* were also found in ASD patients (Marshall et al., 2008; Zahir et al., 2008; Glessner et al., 2009). Interestingly, NRXN1 deletions also confer risk for schizophrenia pointing to an overlap between the two neurodevelopmental disorders (Betancur et al., 2009; Rujescu et al., 2009).

#### Contactins

The Contactins (CNTNs) are glycosyl phosphatidyl-inositol (GPI) anchored immunoglobulin (Ig) superfamily proteins with diverse functions ranging from myelination (Berglund et al., 1999; Bhat et al., 2001) to synapse formation and plasticity (Betancur et al., 2009). Disruption of *CNTN4* is associated with ASD (Fernandez et al., 2004). Deletion and duplication in *CNTN4* and small deletions near *CNTN3* have been identified in various patients with ASD (Roohi et al., 2009). Since *CNTN3* and *CNTN4* expression and localization overlaps with synaptogenesis in the developing brain, it raises the possibility that mutations or genomic rearrangements in these genes seen in ASD could be attributed to altered synapse formation and function.

#### Contactin-associated protein like 2

Contactin-associated protein like 2 (CNTNAP2) is a member of Neurexin superfamily and is a locus that is significantly associated with susceptibility for ASD (Alarcon et al., 2008; Arking et al., 2008). *CNTNAP2* encodes CASPR2, a multidomain transmembrane protein that is best known for clustering potassium channels at the juxtaparanodes in myelinated axons (Poliak et al., 2003). CNTNAP2 localizes at high levels in human fetal brain prior to myelination (Abrahams et al., 2007). It also shows a distribution gradient as frontal cortical enrichment in the developing human brain, indicative of a role in patterning circuits that underlie higher cognition and language. Thus, *CNTNAP2* might play a role in the developing brain regions that are likely to be affected in ASD. A recessive frameshift mutation in *CNTNAP2* was identified in individuals with cortical dysplasia focal epilepsy syndrome, a congenital disorder, where majority of individuals displayed characteristic features of ASD (Strauss et al., 2006). In addition, other studies that attribute *CNTNAP2* to ASD include rare single base pair mutations and common variations in the *CNTNAP2* locus identified in patients with ASD (Alarcon et al., 2008; Arking et al., 2008; Bakkaloglu et al., 2008; Falivelli et al., 2012). Recent phenotypic characterization of *Cntnap2* mutant mice revealed deficits in the three core ASD behavioral domains with hyperactivity and epileptic seizures (Penagarikano et al., 2011). These mutant mice also showed neuronal migration abnormalities, a significant reduction in the number of interneurons, and abnormal neuronal network activity before the onset of seizures. Most importantly, treatment with the FDA-approved drug risperidone led to amelioration of the repetitive behaviors in the mutant mice further demonstrating a functional role for CNTNAP2 in neuronal development and opening of new avenues for therapeutic intervention in ASD (Penagarikano et al., 2011).

#### NrCAM

NrCAM is a CAM that has gene homology to NgCAM and is capable of homophilic cell adhesion as well as heterophilic interactions with other non-NrCAM molecules such as Contactin-1, Contactin-2/TAG1, and Neurofascin (Suter et al., 1995; Volkmer et al., 1996; Pavlou et al., 2002). More recently, association analysis has linked NrCAM to ASD (Sakurai et al., 2006). This study showed over transmission of particular haplotypes of NrCAM that modulate NrCAM expression in the brain, are associated with a specific subset of autism with a severe obsessive–compulsive behavior. Several single nucleotide polymorphisms (SNPs) in the NrCAM gene were also found to be associated with autism (Marui et al., 2009) further underscoring NrCAM as a strong candidate gene in ASD.

#### Cadherins

Cadherins (CDH) and protocadherins (PCDH) include a large family of CAMs, a number of which are required for synaptic formation and function (Weiner et al., 2005; Arikkath and Reichardt, 2008). CDHs mostly undergo homophilic cell adhesion and are involved in intracellular signaling pathways associated with neuropsychiatric disorders. Many of the CDHs have specific spatio-temporal expression patterns in the brain and loss of CDHs leads to altered functional connectivity and neuronal information processing in human brain (Redies et al., 2012). Recent studies have identified *de novo* translocation deleting *CDH18* in ASD (Marshall et al., 2008). This study also reported CNVs associated with *PCDH9* gene in ASD. Homozygous deletions in *PCDH10* have also been shown in autistic children (Morrow et al., 2008). Other CDHs and PCDHs are disrupted in disorders related to mental retardation and intellectual disabilities (Weiner and Jontes, 2013).

### ION CHANNELS

Ion channels are essential for regulating axonal conduction of electrical activity and maintaining the optimum level of excitability within the nervous system. Recent studies linked neuronal excitation alterations with ASD pointing to a potential role for ion channels in the etiology of ASD. Mutations in calcium, sodium, and potassium ion channels seem to enhance neuronal excitability. ASD-linked ion channel mutations involve the *SCN1A* (Na_v_1.1), *CACNA1C* (Ca_v_1.2), *KCNMA1* (BK Ca^2^^+^), and *KCNJ10* (Kir4.1) channels (Ji et al., 2009; Liao and Soong, 2010; Li et al., 2011; Sicca et al., 2011).

#### Na_v_1.1

*SCN1A* encodes the alpha subunit of the sodium channel type 1 (Na_v_1.1) which belongs to the voltage-gated sodium channel family necessary for axonal conduction and action potential propagation. These transmembrane proteins possess a large pore-forming alpha subunit and two auxiliary beta subunits. This organization is important for allowing sodium ions to move through the axonal membrane to initiate and propagate action potentials. Recently, *SCN1A* has emerged as the most important gene in epilepsy (Mulley et al., 2005). More that 70% of individuals with epileptic encephalopathy posses a mutation in the region encoding SCN1A causing severe myoclonic epilepsy in infancy, also known as Dravet syndrome (DS; Harkin et al., 2007). This disorder is often accompanied by certain behavioral abnormalities such as hyperactivity, sleep-disorder, anxiety, attention deficit, impaired social interactions, restricted interests, and severe cognitive defects (Weiss et al., 2003; Ramoz et al., 2008; O’Roak et al., 2012). Such behaviors are very similar to those observed in patients with ASD and emerging evidence has linked SCNA1 and ASD (Li et al., 2011). Researchers found that mice with a loss of function mutation for *SCN1A* phenocopy DS and show autistic-like behaviors (Han et al., 2012). It was suggested that the autism-related traits in DS mice might be caused by a decrease in inhibitory neurotransmission in GABAergic interneurons due to SCN1A haploinsufficiency providing further evidence that impaired GABAergic signaling may underlie ASD (Chao et al., 2010).

#### Ca_v_1.2

The calcium channels, voltage-dependent, L type, alpha 1C subunit, also known as Ca_v_1.2 encoded by the gene *CACNA1C* has been implicated in ASD. Ca_v_1.2 channels are important in the activation of transcription factors and play a key role in promoting neuronal survival and dendritic arborization (Krey and Dolmetsch, 2007). A mutation in the G406R region of the CACNA1C gene is known to cause TS, a rare genetic disorder that results in malformations of multi-organ systems, neurological and developmental defects, and autism (Liao and Soong, 2010). The mutation in this region causes prolonged inward current and has dramatic effects on calcium channel inactivation (Splawski et al., 2004). The cellular and molecular consequences of this mutation are not yet known. Future studies should address the physiological relationship between calcium channel inactivation and ASD.

#### Kir4.1 and BKCa

Recent studies have linked potassium channel proteins Kir4.1 and BKCa to ASD. Mutational screens identified several missense mutations in the *KCNJ10* region encoding the potassium ion channel Kir4.1. This ATP sensitive inward rectifier type potassium channel is characterized by having a greater tendency to allow potassium ions to flow into the cell and is suggested to be responsible for the buffering action of glial cells in the brain. Individuals who possess a mutation in the region show symptoms consistent with the DSM-IV-TR criteria for ASD along with seizure and intellectual disability (Sicca et al., 2011). BKCa is a potassium channel known for its large conductance of potassium ions across cell membranes. The gene *KCNMA1* encodes BKCa. which is thought to function as a synaptic regulator of neuronal excitability which seems to be disrupted in patients with ASD (Laumonnier et al., 2006). Disruption of this gene caused a decrease in BKCa channel activity and haploinsufficiency in Autism patients further implicating excessive ion channel activity to ASD (Ji et al., 2009).

### SCAFFOLDING PROTEINS

Scaffolding proteins are essential molecules of the synaptic architecture. They are enriched in post-synaptic densities (PSDs) and function in synapse biogenesis by trafficking and anchoring synaptic proteins and clustering of membrane-associated proteins. Most importantly, the scaffolding proteins serve to link post-synaptic receptors with their downstream signaling components and regulate cytoskeletal dynamics (Verpelli et al., 2012).

#### Shank

Shank protein family is one such synaptic scaffolding family of proteins that includes Shank1, Shank2, and Shank3. They have multiple protein–protein interaction domains and are also known as proline-rich synapse-associated proteins (ProSAPs). Shank proteins are enriched in PSDs and stabilize the PSD-95/SAPAP/Shank/Homer complex (Tu et al., 1999; Sala et al., 2001). Additionally, Shank interacts with NMDA receptors/PSD-95/GKAP complex and actin regulatory protein, Cortactin (Naisbitt et al., 1999; refer **Figure 1**). Strong genetic and molecular evidence has linked *SHANK2* and *SHANK3* to the development of ASD phenotypes.

#### SHANK2

Mutations in *ProSAP1/Shank2* gene result in an upregulation of glutamate receptors in certain brain regions, an increase in Shank3 at the synapse, and a decrease in dendritic spine morphology and synaptic transmission (Schmeisser et al., 2012). *ProSAP/Shank2* mutants also display behavioral phenotypes that are consistent with those seen in ASD. *Shank2* mutant mice are hyperactive, exhibit repetitive grooming, and have impairments in social and vocal behaviors (Schmeisser et al., 2012). Such phenotypic manifestations are linked to the reduction of NMDAR function that results from the absence of the Shank2 protein (Won et al., 2012). Human studies using microarray analyses have identified several variants in *SHANK2* that are associated with ASD and mental retardation (Berkel et al., 2010).

#### SHANK3

Shank3 is an important member of Shank family of proteins and interacts with NLGN (Gerrow et al., 2006) to play a key role in spine morphogenesis and synaptic plasticity (Sala et al., 2001). Recent studies on Shank3 using knockout mice suggest its involvement in the regulation of glutamatergic synapse size, shape, and structure (Jiang and Ehlers, 2013). In *Shank3* knockout mice, synaptic ultrastructure is compromised. Overall, shank3 loss leads to a reduction in spine volume, decreased PSD thickness, and loss of dendritic spines (Bozdagi et al., 2010; Peca et al., 2011a; Wang et al., 2011; Jiang and Ehlers, 2013). Furthermore, *Shank3* knockout mice show abnormal social behaviors, communication patterns, repetitive behaviors, and impairments in learning and memory (Bozdagi et al., 2010; Peca et al., 2011a; Wang et al., 2011).

There is growing evidence of the involvement of Shank3 in ASD. Molecular characterization of individuals with 22q13.3 deletion syndrome that display autism behavior identified a deletion disrupting *Shank3* among other genes (Wilson et al., 2003). Haploinsufficiency of *Shank3* has been confirmed to account for 22q13 deletion phenotype of developmental and speech delays (Durand et al., 2007). Other studies that attributed a role of Shank3 in ASD include identification of *de novo* splice site mutation in ASD (Gauthier et al., 2009). More recently, Shank3 mutations identified in patients with ASD show a modification in dendritic spine induction and morphology as well as actin accumulation in spines affecting growth cone motility (Durand et al., 2007). Furthermore, a microdeletion in *Shank1* locus has been discovered using microarray analysis in individuals with ASD (Sato et al., 2012). Recent studies have further uncovered the functional role of Shank3 as *Shank3* duplication in mice leads to hyperactivity and spontaneous seizures much like human subjects who have small duplications in the *SHANK3* locus. These recent studies further underscore the function of Shank3 in neuronal function and possibly in the maintenance of a balance between the excitatory and inhibitory (E/I) synaptic mechanisms (Han et al., 2013).

#### SynGAP1

*SynGAP1* encodes the RAS GTPase-activating protein (GAPs) which is a critical component of the PSD. At the PSD, SynGAP1 regulates synapse development and maintenance of proper synaptic function. It is known to interact with PSD-95 and colocalizes with excitatory NMDA receptor complexes (Chen et al., 1998). SynGAP is shown to play a critical role in the PSD during early postnatal development as SYNGAP1 knockout mice for die during early development (Kim et al., 2003). Furthermore, mice heterozygous for SynGAP1 show impairments in learning and memory consistent with its involvement in NMDA receptor complexes (Komiyama et al., 2002). In humans, sequencing of the *SYNGAP1* locus revealed mutations linked to non-syndromic mental retardation evidencing the importance of SynGAP in synaptic plasticity and learning (Hamdan et al., 2009). SynGap1 was recently implicated ASD because many of its key interacting partners including PSD-95/DLG4, SAP-102/DLG3, PSD-93/DLG2, Neurexins, and Neuroligins have previously been associated with ASD (van de Lagemaat and Grant, 2010). Recent evidence suggests that SynGAP may play a crucial role in controlling the E/I balance in cortical neurons through the regulation on ERK signaling pathways (Wang et al., 2013). This is interesting because the E/I balance seems to be altered in individuals with ASD (Eichler and Meier, 2008; Won et al., 2013). Further characterization of SynGAP as a regulator of synaptic function will provide additional insight into its involvement in ASD.

### CYTOSKELETAL PROTEINS

A set of cytoskeletal proteins is also mutated in individuals with ASD. These include factors regulating dynamics of actin cytoskeleton, such as GAPs and guanosine exchange factors (GEFs; Newey et al., 2005). Rare non-synonymous variants in *cAMP-GEFII* are among candidate genes for autism in chromosome 2q (Bacchelli et al., 2003). Mutations in tumor suppressor genes *TSC1* and *TSC2* are also linked to ASD as the mutant proteins are thought to perturb cytoskeletal dynamics and dendritic spine structure in mutant animals (Folstein and Rosen-Sheidley, 2001). More recently, microtubule associated protein, KATNAL2, has emerged as a credible risk factor for ASD (Neale et al., 2012).

Apart from CAMs, scaffolding and cytoskeletal proteins, a host of other receptors, transporters and channel proteins are known to contribute toward the etiology of ASD (summarized in **Table 4**). Discovery of more and more genes and genetic pathways are expanding the genetic landscape of ASD. It is interesting to note that these genes include chromatin modifiers, DNA binding proteins, ion channels, and neurotransmitter receptors (State and Sestan, 2012). Recently, CNVs in new candidate genes within GABAergic signaling and neural development pathways associated with ASD were identified using genome wide SNP array (Griswold et al., 2012). These genes include an allosteric binder of GABA receptor, DB1, the GABA receptor-associated protein, GABARAPL1 and a post-synaptic GABA transporter, SLC6A11. Other genes contributing to the genetics of ASD include glutamate receptors, such as GRID1, GRIK2, and GRIK4, synaptic regulators, such as SLC6A8 and SYN3, and transcription factors, such as Engrailed 2 (EN2; Griswold et al., 2012).

### SIGNALING PATHWAYS

Signaling pathways are a complex system of communication within cells that function to organize cellular activities. Signaling pathways and cascades have long been implicated in many disease models. Understanding signaling pathways may play a central role in developing pharmacological or other agents to better treat disease. Currently, disruptions in signaling pathways are being linked to the development of ASD phenotypes. One such pathway connected to ASD is the mammalian target of rapamycin (mTOR) pathway.

### mTOR PATHWAY

Mammalian target of rapamycin is a serine/threonine protein kinase involved in the regulation of cell proliferation, cell growth, cell survival, protein synthesis, and transcription. The mTOR signaling cascade plays a very important role in synapse protein synthesis and several studies have linked this pathway to ASD (Hay and Sonenberg, 2004; Hoeffer and Klann, 2010). Many of the signaling components of the mTOR cascade are located at the synapses where they have been shown to regulate dendritic spine morphology and synaptogenesis (Kumar et al., 2005). Mutations in the proteins known to inhibit mTOR signaling including NF1, PTEN, TSC1, and TSC2 are all linked to neurological disease and autistic-like behavioral phenotypes (Williams and Hersh, 1998; Butler et al., 2005; Won et al., 2013). Furthermore, mutations in the downstream targets of the mTOR signaling cascade have been identified in patients with ASD. The mTOR signaling cascade works by the phosphorylation of 4E-BP by mTOR. This causes 4E-BP to dissociate from the eIF4E initiation factor resulting in cap-dependent translation and elongation of mRNA (Laplante and Sabatini, 2009; Wang and Doering, 2013). Genomic sequence analyses of the eIF4E promoter region identified a SNP in autism patients that enhanced the promoter activity of eIF4E (Neves-Pereira et al., 2009). Additionally, 4E-BP knockout mice as well as mice with an overexpression of eIF4E show autistic like behaviors, enhanced translational of Neuroligins, and disruptions in the E/I balance (Gkogkas et al., 2013; Won et al., 2013). Analyses of monogenetic sources of ASD found that approximately 8–10% of all ASD are involved in regulation of the mTOR pathway (Moldin et al., 2006; Kelleher and Bear, 2008; Hoeffer and Klann, 2010). Of those, 1–2% of ASD cases result due to a mutation in the gene encoding PTEN, an upstream member of the mTOR pathway (Hoeffer and Klann, 2010). The other upstream members, TSC1 and TSC2, form a heterodimer complex. Mutations in the genes encoding this complex cause TSC which is defined clinically by the appearance and growth of benign hamartomas throughout the body and brain (Smalley, 1998). TSC patients suffer from mental retardation and epilepsy. Recent studies show that 25–50% of patients with TSC show behaviors that are consistent with ASD behavioral phenotypes (Hoeffer and Klann, 2010). Pharmacological manipulations to identify therapeutic targets that may be enhancers and suppressors of mTOR signaling cascades and mRNA translation are currently being explored to combat some of the phenotypic manifestations associated with ASD (Carson et al., 2012). Further studies into the downstream and upstream targets of the mTOR signaling cascade will provide additional insights into the functional relationship between the mTOR pathway and ASD.

## CONCLUDING REMARKS

Current efforts to identify the constellation of genes that confer the characteristic phenotypic manifestations within the autism spectrum have improved our understanding of this complex disorder. While modeling mutations in experimental animal model systems will highlight the underlying disruptions in conserved signaling pathways, the daunting task will still be to establish ASD-specific phenotypes at the molecular, cellular and neural circuit levels. The staggering number of genes already discovered in ASD holds the promise to translate the knowledge into designing new therapeutic interventions. The very interesting and equally challenging observation from the recent genetic studies has been a high degree of overlap of risk factors for various neurodevelopmental disorders, such as ASD, epilepsy, and schizophrenia. This pattern of overlap provides the feasibility to address which genes and genetic pathways intersect and specify the spatio-temporal sequence of events that occur within the developing human brain. The recent advent of comprehensive maps of spatio-temporal gene expression in the human brain (Kang et al., 2011) will greatly help toward providing a powerful developmentally informed approach to studying disorders such as ASD.

Although concerted efforts from studies of human clinical ASD populations and various ASD-related animal models have provided a better understanding of the genetic, molecular, and circuit level aberrations in ASD, several intriguing, yet significant questions still remain. For instance, how can the compound effects of genetics, epigenetics, and environment be consolidated in understanding ASD pathogenesis? What other events play a role in determining the appearance and trajectory of ASD symptoms? How do the majority of the genetic susceptibility loci in ASD affect synapse assembly, maintenance and functional modulation? Finally, how would the future treatments and interventions be designed and organized to accommodate the ever-changing genetic landscape of ASD?

## Conflict of Interest Statement

The authors declare that the research was conducted in the absence of any commercial or financial relationships that could be construed as a potential conflict of interest.
